# Analysis of the role of the *Cronobacter sakazakii* ProP homologues in osmotolerance

**DOI:** 10.1186/1757-4749-6-15

**Published:** 2014-05-24

**Authors:** Audrey Feeney, Christopher D Johnston, Rodney Govender, Jim O’Mahony, Aidan Coffey, Roy D Sleator

**Affiliations:** 1 Department of Biological Sciences, Cork Institute of Technology, Rossa Avenue, Bishopstown, Cork, Ireland

**Keywords:** Osmolytes, Proline, Osmotolerance, Stress, *Cronobacter*

## Abstract

Bacteria respond to elevated osmolality by the accumulation of a range of low molecular weight molecules, known as compatible solutes (owing to their compatibility with the cells' normal physiology at high internal concentrations). The neonatal pathogen *Cronobacter sakazakii* is uniquely osmotolerant, surviving in powdered infant formula (PIF) which typically has a water activity (a_w_) of 0.2 – inhospitable to most micro-organisms. Mortality rates of up to 80% in infected infants have been recorded making *C. sakazakii* a serious cause for concern. In silico analysis of the *C. sakazakii* BAA-894 genome revealed seven copies of the osmolyte uptake system ProP. Herein, we test the physiological role of each of these homologues following heterologous expression against an osmosensitive *Escherichia coli* host.

## Introduction

*Cronobacter spp.* (previously *Enterobacter sakazakii*) are Gram-negative, motile, facultatively anaerobic foodbourne pathogens belonging to the Enterobacteriacea family [1]. The Cronobacter genus, which has become increasingly diverse in recent years, comprises ten species: *Cronobacter sakazakii*, *Cronobacter malonaticus*, *Cronobacter turicensis*, *Cronobacter universalis*, *Cronobacter muytjensii*, *Cronobacter dublinensis*, *Cronobacter condiment, Cronobacter pulveris, Cronobacter helveticus and Cronobacter zurichensis*[2,3]. *Cronobacter sakazakii* is an opportunistic pathogen capable of causing severe clinical symptoms in adults, infants and elderly patients (FAO/WHO 2008). The most at risk group are pre-term, low birth weight neonates who can suffer life-threatening necrotizing enterocolitis, meningitis and septeceamia, with resulting mortality rates of 40-80% [4]. In a recent study multilocus sequence typing revealed that the majority of meningitis cases in neonates are caused as a result of infection with one clonal lineage, *C. sakazakii* ST4 clonal complex [2]. While a significant proportion of infants succumb to the infection within days of birth [5], survivors often suffer severe neurological damage [6].

While studies have identified plant material (in particular vegetables, fruits, cereals and rice) as the most likely source, foods such as wheat, cheese, meat, herbs and spices have also been found to harbour the pathogen [7]. However, it is the presence of *C. sakazakii* in powdered infant formula (PIF) which is cause for most concern (FAO/WHO 2008). Indeed, PIF has been epidemiologically linked with several major outbreaks of *C. sakazakii* infection [8]. Although isolated at low levels, persistence of the pathogen for extended periods, in some cases for up to 2.5 years [9] makes *C. sakazakii* a particularly formidable pathogen. Given that pasteurisation has been shown to effectively inactivate *C. sakazakii*, the presence of the organism in PIF can be attributed to contamination resulting from the addition of contaminated ingredients and/or the use of non-sterile equipment either during processing or reconstitution [10].

Survival of *C. sakazakii* in PIF, which has an a_w_ of ~ 0.2 (significantly below the normal cut off of 0.4 for canonical microbial growth), suggests that the pathogen is uniquely armed with an extensive array of stress survival mechanisms necessary to overcome this otherwise inhospitable environment [11]. Indeed, a previous *in silico* analysis of *C. sakazakii* BAA-894 (a strain originally isolated from powdered infant formula [12]) revealed 53 putative osmotolerance loci, including both hyper- and hypo-osmotic stress response systems. Interestingly, while *C. sakazakii* harbours homologues of all the principal osmotolerance loci of *Escherichia coli*; a key distinguishing feature is that *C. sakazakii* possesses multiple copies of certain osmotolerance mechanisms; including seven copies of the *E. coli proP* homologue, each of which potentially encodes a separate osmolyte uptake system.

Herein, we describe the functional analysis of the putative osmotolerance loci ESA_02131, ESA_01706, ESA_04214, ESA_pESA3p05450, ESA_00673 and ESA_03328, when heterologously expressed against *E. coli* MKH13 (an osmotically sensitive mutant). Furthermore, given that the *C. sakazakii* osmotic stress response is likely regulated at the level of DNA expression [11], we analysed the transcriptional response of each of the *proP* homologues following osmotic up-shock.

## Materials and methods

### Bacterial strains, plasmids and growth conditions

Bacterial strains and plasmids used in this study are listed in Table 1. *C. sakazakii* BAA-894 and *E. coli DH5α* clones were grown at 37°C in LB broth (Sigma-Aldrich Co.). *E. coli MKH13* strains were grown in LB or M9 minimal medium containing 0.5% glucose, 0.04% arginine, 0.04% isoleucine, 0.04% valine (Sigma-Aldrich Co.). After filter sterilization, proline or glycine betaine (Sigma-Aldrich Co.) was added to M9 to a final concentration of 1 mM. Ampicillin was made up as a concentrated stock solution and added to media at the required levels. Medium osmolarity was adjusted using NaCl.

**Table 1:** Bacterial strains and plasmids

**Strain or plasmid**	**Relevant genotype or characteristics**	**Source or reference**
**Plasmids**		
pUC18	Amp^r^, lacZ', pMB9 replicon	[13]
pUC18::ESA_02131	pUC18 with ESA_02131 gene under control of native promoter	This work
pUC18::ESA_01706	pUC18 with ESA_01706 gene under control of native promoter	This work
pUC18::ESA_04214	pUC18 with ESA_04214 gene under control of native promoter	This work
pUC18::ESA_pESA3p05450	pUC18 with ESA_pESA3p05450 gene under control of native promoter	This work
pUC18::ESA_01226	pUC18 with ESA_01226 gene under control of native promoter	This work
pUC18::ESA_00673	pUC18 with ESA_00673 gene under control of native promoter	This work
pUC18::ESA_03328	pUC18 with ESA_03328 gene under control of native promoter	This work
**Strains**		
*Cronobacter sakazakii* BAA-894	*C.sakazakii* strain isolated from powdered formula associated with neonatal intensive care unit	[12]
*Escherichia coli* DH5α	Intermediate cloning host.supE44 ΔlacU169(80lacZΔM15)R17 recA1 endA1 gyrA96 thi-1 relA1.	Invitrogen.
MKH13	MC4100Δ(putPA)101Δ(proP)2Δ(proU)	[15]
MKH13 pUC18::ESA_02131+	Host strain harbouring pUC18: ESA_02131 plasmid. Amp^r^.	This work
MKH13 pUC18::ESA_01706+	Host strain harbouring pUC18: ESA_01706 plasmid. Amp^r^.	This work
MKH13 pUC18::ESA_04214+	Host strain harbouring pUC18: ESA_04214 plasmid. Amp^r^.	This work
MKH13 pUC18::ESA_pESA3p05450+	Host strain harbouring pUC18: ESA_pESA3p05450 plasmid. Amp^r^.	This work
MKH13 pUC18::ESA_01226+	Host strain harbouring pUC18: ESA_01226 plasmid. Amp^r^.	This work
MKH13 pUC18::ESA_00673+	Host strain harbouring pUC18: ESA_00673 plasmid. Amp^r^.	This work
MKH13 pUC18::ESA_03328+	Host strain harbouring pUC18: ESA_03328 plasmid. Amp^r^.	This work

Amp^r^ - This strain is resistant to ampicillin.

### *In silico* analysis of *C. sakzakii* BAA-894

Putative *proP* loci were identified using a homology based transfer approach, as described previously [11]. Further *in silico* analysis was carried out with the aid of the Lasergene suite of applications (DNASTAR, Madison, USA) and Simplicity [16] (nSilico, Cork, Ireland).

### DNA manipulations and sequence analysis

Genomic DNA was extracted from *C. sakazakii* as described by Sambrook et al. [17]. Plasmid DNA was isolated with the High Pure Plasmid Isolation Kit (Roche Diagnostics). PCR primers (Table 2) were designed for each *proP* homologue based on *C. sakazakii* strain BAA-894 sequence data available from the NCBI database (NC_009778.1). All PCR reactions were carried out using the high fidelity Velocity DNA polymerase Kit (Bioline) in accordance with manufacturer's instructions. The T*a* of each reaction was dependent on the T*m* of individual primers. Negative control reaction mixtures were included with sterile nuclease free water in place of template DNA. Restriction enzymes and T4 DNA ligase were purchased from Roche Diagnostics (Mannheim, Germany). Each restriction digest and T4 ligation reaction was carried out as per the manufacturer's instructions within 0.2 ml PCR tubes (Sarstedt) in a thermocycler block to ensure optimal temperature conditions. Plasmid pUC18 was used as the cloning platform; with individual vectors being constructed for the expression of each gene under the control of its own native promoter. The integrity of each *C. sakazakii* gene was confirmed by sequencing (MWG Operon, Germany and GATC, Germany). Electrocompetent *E. coli* DH5α were obtained from New England Biolabs and *E. coli* MKH13 was made electrocompetent using the method outlined by Sambrook and Russell [17]. Electrotransformation was carried out using standard methods.

**Table 2:** Primers

***Primer name***		***Primer sequence (5' to 3')***	***Length***	***Characteristics***
ESA_02131	F	CATCGGCCGACAGGCCAGTCAATGAATGATGC	32	Eag1cut site
	R	CATTCTAGAGAGTACAACGGAATGCGGGG	29	Xba1 cut site
ESA_01706	F	CATTCTAGAGTCGGGCGGCTCTTTATCTGG	30	Xba1 cut site
	R	CATGGATCCTTGACCAGATGACGCAGTCTTTC	32	BamH1 cut site
ESA_04214	F	CATGAATTCGTCTCTTTCTGTGCCAACTATCTGC	34	EcoR1 cut site
	R	CATTCTAGACTACCTGACGCGTACCCTGTATATC	34	Xba1 cut site
ESA_pESA3p05450	F	CATGAATTCATCATCTCTACACGCTGCCTTCTG	33	EcoR1 cut site
	R	CATTCTAGATCTCCACCTGCGCCTCTATC	29	Xba1 cut site
ESA_01226	F	CATGAATTCCAGTGCGCCGGAGCTTTTCG	29	EcoR1 cut site
	R	CATTCTAGAGGGCTGTCGGTTGACGAAATTAAAC	34	Xba1 cut site
ESA_00673	F	CATGAATTCTAAAAGCGAAATCCTCCCGTACTGGC	35	EcoR1 cut site
	R	CATGGATCCCCTGCAAAGCATCGCCGATTACC	32	BamH1 cut stie
ESA_03328	F	CATTCTAGATCGCTATCGCTGACCGTGAAATG	32	Xba1 cut site
	R	CATGGATCCTGCTGAACGAACAGTATGGCCG	31	BamH1 cut site
pUC18 MCS Check	F	CATTAG CTC ACT CAT TAG GCA CC	20	pUC18 insert check
	R	CATTGT AAA ACG ACG GCC AGT G	19	pUC18 insert check
RTESA_02131	F	GCTGGCGTGTATCGGTCT	18	Probe 31
	R	CTC GGC ATA TAA GTA AGC AGC AT	23	Probe 31
RTESA_01706	F	CGA CGG TCA TTC TGC TCA C	19	Probe 32
	R	AGC AGG CCA ATC TGA TGG TA	20	Probe 32
RTESA_04214	F	GCA TAA ACG CGC CCT GTA	18	Probe 18
	R	TTA GCG AAG AAG AAG CCG ATA	21	Probe 18
RTESA_01226	F	GGG TAT CAG GTG GCA AGC	18	Probe 70
	R	GTA AAT CGC GAC CGT ATG C	19	Probe 70
RTESA_00673	F	CCG GAC AGA TAA ACC GTC AC	20	Probe 11
	R	GAC GAG GCA CCG ACA ATC	18	Probe 11
RTESA_03328	F	CGT GCC GTT CGT AAT GGT	18	Probe 29
	R	AAC GAA ATG CCG GTA AAG C	19	Probe 29
RTESA_pESA3p05450	F	TGG TGG CGA TTT CCA ACT	18	Probe 71
	R	CCC GAA ACG GTC AGA AAG	18	Probe 71
RT16S	F	TGT AGC GGT GAA ATG CGT AG	20	Probe 65
	R	AGC GTC AGT CTT CGT CCA G	19	Probe 65

### Gene expression analysis (qRT-PCR)

Total RNA was isolated (Roche High Pure RNA Isolation Kit) from *C. sakazakii* grown in a shaking incubator at 37°C prior to osmotic shock and up to 2 hours post shock with 6% NaCl. RNA quantity and quality was confirmed by microphotometry (Nanodrop, De, USA). Complementary DNA (cDNA) was synthesised using 1 μg total RNA incubated with 50pM of random hexamer primers followed by a 20 μl reverse transcription reaction using the Bioloine cDNA synthesis kit, as per manufacturer’s instructions (Bioline). The amplification conditions used were 42°C for 1 hour, 70°C for 15 min and 4°C for 5 min. PCR primers and probes were designed using the Universal Probe Library Assay Design Centre (http://lifescience.roche.com/shop/home) and are summarised in Table 2. Optimisation of the gene expression assay was carried out on each primer/probe set by varying the concentration of either primer or probe to achieve the best signal. Based on these results, individual primer/probe mixes were made for each gene of interest. Amplification reactions contained 5 μl cDNA, 10 μl of the Light Cycler 480 probes Master 2x conc (Roche) and 2 μl primer/probe mix in a total reaction of 20 μl. All reactions were performed in triplicate using 96 well plates on the Roche LightCycler 480 Real-Time PCR system. Thermal cycling conditions were as recommended by the manufacturer. The 2^Δ Δ^CT method was used to calculate relative changes in gene expression determined from these real time quantitative PCR experiments [18].

### Physiological analysis of *E. coli* MKH13 clones in media supplemented with NaCl

Each *E. coli* MKH13 clone expressing a different *proP* homologue (ESA_02131, ESA_01706, ESA_04214, ESA_pESA3p05450, ESA_00673 or ESA_03328) was grown overnight at 37°C in 10mls of LB, or minimal media supplemented with the required components. The cells were pelleted by centrifugation at 5,000 g, washed and resuspended in 200 μl ringers. The cell suspension was added to the appropriate filter sterilized media with varying concentrations (0-10%) of added NaCl. Growth was monitored in the relevant media over a 48 hour period and the optical density (OD) was measured at 600 nm. Triplicate readings were taken and graphs were constructed using SigmaPlot 11.0.

## Results

### Functional complementation of *E. coli* MKH13 with *proP* homologues

Each of the *proP* homologues were amplified using primers with engineered cut sites flanking the gene of interest. PCR products were digested and ligated to similarly digested pUC18 plasmid. Each pUC18::*proP* plasmid was transformed to MKH13 following passage through an intermediate cloning host (DH5α). The osmosensitive *E. coli* strain MKH13 was used to functionally screen each of the *proP* homologues for their osmotolerance potential. Unlike the parent strain, MC 4100, *E. coli* MKH13 is deficient in the transport systems PutP, ProP and ProU; rendering it incapable of growth in high osmolarity media (≥4% added NaCl). Six of the seven *proP* homologues were successfully transformed into *E.coli* MKH13 (ESA_01226 alone failed to be transformed and expressed in *E.coli* MKH13, despite repeated transformation attempts). Successful transformants were screened for osmotolerance by plating on LB agar and on defined media agar plates (supplemented with proline or glycine betaine to a final concentration of 1 mM) containing 4-10% added NaCl. No colonies appeared following a control transformation with pUC18 alone, while transformation efficiencies of ~60 CFU/μg DNA were achieved for each of *proP* homologue constructs, with colonies appearing within 72 hours at 37°C. Plasmids were extracted and retransformed into *E. coli* MKH13 to confirm complementation and inserts were confirmed by PCR and sequence analysis.

### *proP* expression analysis in *C. sakazakii* BAA-894

**Figure 1:**
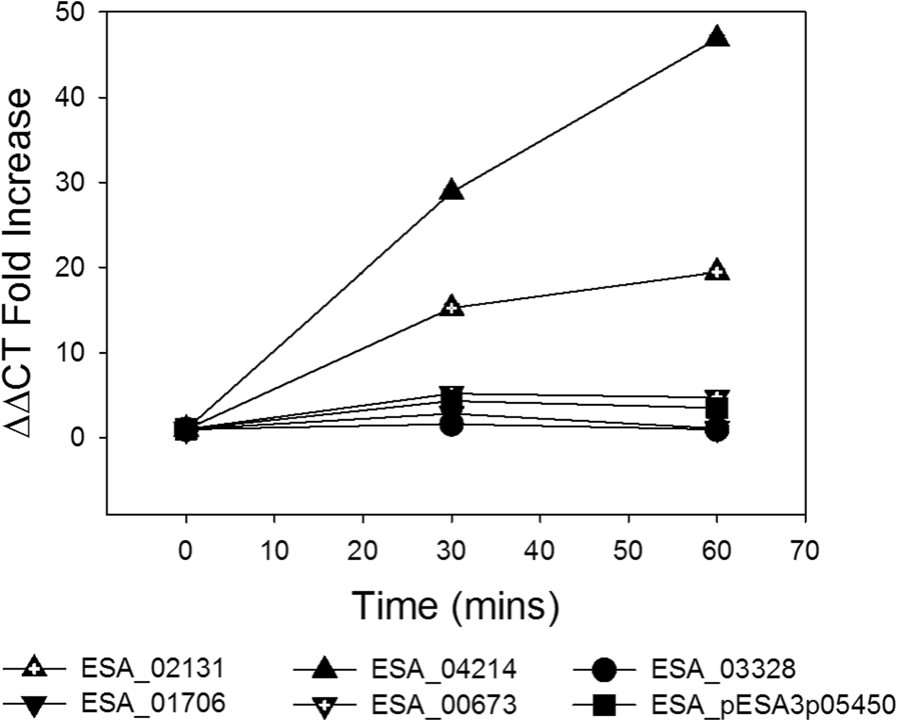
**Gene expression analysis of each *proP *** **homologue in *C. sakazakii *** **BAA-894 when subject to osmotic stress with 6% added NaCl.**

The level of gene expression was measured relative to the constitutively expressed 16 s gene (ESA_03798). *E. coli* MKH13 expressing *proP* homologues ESA_02131, ESA_01706, ESA_04214, ESA_00673, ESA_03328 and ESAp_3p05450 exhibited an increase in expression levels in complex media (LB) following osmotic up-shock with 6% NaCl (Figure 1). Strains expressing ESA_02131 and ESA_04214 exhibited the highest increase in expression 1 hour post initial osmotic shock (19-fold and 47-fold increase respectively). However, maximum levels of expression were reached 30 minutes after initial osmotic shock in *E. coli* MKH13::01706 (3 fold increase), *E. coli* MKH13::00673 (5 fold increase), *E.coli* MKH13::03328 (2 fold increase) and *E. coli* MKH13::pESA3p05450 (4 fold increase) (Table 3). This suggests a biphasic response at the transcriptional level; a rapid low level up regulation followed by a slower high level response.

**Table 3:** Molecular and physiological comparison of each ProP homologue

**Gene locus tag**	**Identity (%)**	**Fold increase in mRNA transcript**	**Complex media**		**Defined media supplemented with proline**		**Defined media supplemented with glycine betaine**	
			**Maximum OD @ 600 nm**	**Growth rate (hr**^**-1**^**)**	**Maximum OD @ 600 nm**	**Growth rate (hr**^**-1**^**)**	**Maximum OD @ 600 nm**	**Growth rate (hr**^**-1**^**)**
ESA_02131	90	19.472	0.668	0.013	0.219	0.007	0.585	0.017
ESA_01706	40	2.854	0.259	0.000	0.205	0.008	0.358	0.011
ESA_04214	33	46.851	0.240	0.001	0.178	0.000	0.240	0.000
ESA_03328	30	1.598	0.255	0.003	0.261	0.021	0.397	0.021
ESA_00673	29	5.193	0.505	0.013	0.125	0.000	0.273	0.000
ESA_pESA3p05450	29	4.327	0.291	0.003	0.170	0.003	0.235	0.002

The % identity values were obtained in a previous study [11] and determined the homology of the above *C. sakazakii* putative *proP* genes relative to the well characterised *E. coli proP.* Growth rate, OD and transcription values shown here were obtained when growth was monitored in 6% added NaCl.

### Physiological analysis of *proP* homologues expressed in *E. coli* MKH13

Physiological analysis of each *E. coli* MKH13 strain transformed with a *proP* homologue was performed to determine if each homologue conferred osmotolerance on the heterologous host. Growth of *E. coli* MKH13 was monitored over a 48 hour period in complex (LB) and defined media with varying concentrations of NaCl (0-10% added NaCl). *E. coli* MKH13 expressing the empty pUC18 vector was used as a negative control; failing to grow at NaCl concentrations ≥4% added NaCl.

#### a) *Growth in complex media at elevated osmolarity*

Each of the six strains expressing a *proP* homologue grew in LB broth supplemented with ≥ 4% added NaCl, with the control strain (*E. coli* MKH13::pUC18) exhibiting no growth. At the most discriminatory salt concentration (6% added NaCl) growth was observed in five of the strains tested but to varying degrees (Table 3). However, above 6% NaCl only strains expressing ESA_00673, ESA_02131 and ESA_pESA3p05450 demonstrated an ability to grow. ESA_00673 conferred the highest osmotolerance with a growth rate of 0.007 h^-1^ at 8% NaCl and a maximum OD of 0.57 @ 600 nm was recorded after 12 hours incubation at 37°C. The next most osmotolerant strains in complex media at elevated osmolarity were those expressing ESA_02131 and the plasmid encoded ESA_pESA3p05450. Both of these strains grew in LB containing up to 7% added NaCl. At this concentration *E. coli* MKH13 expressing ESA_02131 demonstrated a maximum OD of 0.29 after 22 hours incubation and a growth rate of 0.009 h^-1^. Similarly the strain expressing ESA_pESA3p05450 grew to a maximum OD of 0.27 after 11 hours and reached a growth rate of 0.003 h^-1^. While each of the *proP* homologues tested conferred osmotolerance in *E. coli* MKH13, it was observed that the strains expressing ESA_00673, ESA_02131 and ESA_pESA3p05450 demonstrated the highest levels of osmotolerance in an osmolyte rich environment.

#### b) *Growth in defined media plus proline at elevated osmolarity*

Of the six strains tested, *E. coli* MKH13 expressing ESA_02131, ESA_01706, ESA_03328 and ESA_pESA3p05450 demonstrated osmotolerance (growth above 4% NaCl) in defined media supplemented with proline. Each of the four osmotolerant strains grew to an OD ≥ 0.17 at a NaCl concentration of 9%. *E. coli*MKH13 expressing ESA_02131 demonstrated the highest growth rate of the osmotolerant strains (0.007 h^-1^), reaching a final OD of 0.219 at 6% NaCl (Table 3). While the growth rates of *E. coli* MKH13 expressing ESA_02131 in complex media are higher than those grown in defined media supplemented with proline there is also a significant difference in the maximum OD values recorded, with *E. coli* MKH13::ESA_02131 appearing to favour the osmolyte rich environment over media where proline is the only available compatible solute present.

#### c) *Growth in defined media plus betaine at elevated osmolarity*

While *E. coli* MKH13 expressing ESA_00673 failed to grow above 4% NaCl, each of the five remaining strains demonstrated osmotolerance in defined media supplemented with betaine. *E. coli* MKH13::ESA_00673 also failed to grow above 4% NaCl in defined media supplemented with 1 mM proline but demonstrated an ability to grow in LB supplemented with 8% added NaCl. *E. coli* MKH13::04214 demonstrated a growth rate of 0.013 h^-1^ at 5% NaCl in betaine supplemented media but did not grow above 4% NaCl in proline supplemented media. *E. coli* MKH13 expressing ESA_02131, ESA_01706, ESA_03328 and ESA_pESA3p05450 demonstrated the highest osmotolerance in defined media supplemented with betaine, growing up to 9% NaCl. However, significantly higher growth rates and/or OD values were recorded for *E. coli* MKH13 expressing ESA_02131, ESA_01706 and ESA_03328 when compared to growth in proline supplemented media (Table 3).

## Discussion

The adaptation of bacteria to increased osmotic stress involves the intracellular accumulation of organic compounds called osmolytes or compatible solutes (owing to their compatibility with vital cellular processes at high internal concentrations [19-21]). The main function of compatible solutes is to increase cell turgor, thus counterbalancing the external osmotic upshift and preventing water loss, which left unchecked leads to plasmolysis and ultimately cell death. The principal compatible solutes accumulated by *E. coli* under osmotic stress conditions are proline and betaine [22]. The osmoprotective properties of proline were first described by Christian in 1955 who reported that the addition of this amino acid to media at elevated osmolarity prevented bacterial growth inhibition [23]. Likewise, the trimethylamino acid glycine betaine has been extensively studied for its osmoprotective properties [24]. Betaine and proline uptake in *E. coli* is mediated primarily by the secondary uptake system ProP [25].

Previous *in silico* analysis revealed 53 putative osmotolerance loci on the *C. sakazakii* BAA-894 genome. Most notably, seven homologues of the *E. coli proP* gene were identified, each of which was believed to encode a transmembrane protein capable of transporting proline and/or betaine [11]. Further analysis of each ProP homologue revealed structural similarities to the *E. coli* ProP protein with the gene encoding the most obvious ProP candidate ESA_02131 (based on overall% identity), encoding the characteristic 12 trans-membrane domains, an extended central hydrophilic loop and a carboxy-terminal extension [11,26]. Interestingly, although the remaining six proP homologues exhibit significant sequence identity, they are all 60–70 amino acids shorter than ESA_02131 at the C-terminal end. Furthermore, the central hydrophilic loop of the *E. coli proP* is extended and this is a feature common to the protein encoded by the *C. sakazakii* ESA_02131 gene; however sequence analysis has revealed that although a central hydrophilic loop is present in each of the six additional proP homologues this periplasmic loop is not extended to the same degree as ESA_02131 (7–18 amino acids longer in ESA_02131 than other proP homologues identified).

In the current study we investigated the physiological role, particularly in response to salt tolerance, of the ProP porters identified previously in *C. sakazaki.* Firstly, in order to determine if the *proP* homologues are upregulated at the transcriptional level in response to an osmotic upshift, gene expression analysis was performed using the *C. sakazakii* BAA-894 strain. Interestingly, all six *proP* genes analyzed (ESA_02131, ESA_01706, ESA_04214, ESA_00673, ESA_03328 and ESAp_3p05450) demonstrated increased expression levels relative to the constitutively expressed 16 s gene (ESA_03798) when subject to an osmotic upshift; however expression levels varied significantly amongst the individual *proP* homologues (Figure 1). Surprisingly, the homologue which exhibited the highest level of upregulation following osmotic stress was not ESA_02131, the closest homologue to *E. coli proP* in terms of sequence identity, but rather ESA_04214. If we consider that ProP activity in *E. coli* has been shown to be regulated at the translational level *via* ProQ, this may go some way to explaining this incongruity [27]. ProQ is a cytoplasmic protein which was previously believed to play a role in post-translational modification of ProP; influencing the osmotic activation of this compatible solute uptake protein [28,29]. However, more recently, further analysis into the role of ProQ was warranted by the fact that *proP* transcript levels are unchanged in a *proQ* mutant, ProP expression is affected by deletion of *proQ* and no physical interaction was detected between ProP and ProQ [30]. As a result the role of ProQ was recently clarified as a translational regulator of *proP* mRNA. ProQ is a ribosome association protein, interacting with *proP* mRNA transcribed from both RpoD and RpoS promoters. As yet very little is known about the mechanism of ProQ mediated translation, however a deeper analysis of this protein has revealed a high affinity RNA binding domain and an RNA strand exchange and duplexing domain similar to the translational regulator FinO [27,30]. It is likely that, as with *E. coli*, ProP mediated osmotolerance is also regulated at the translational level in *C. sakazakii*, a factor which may account for our observed disconnect between transcriptional data and observed osmotolerance phenotype as outlined below. In support of this proposal, we have identified a ProQ homologue (ESA_01419) on the *C. sakazakii* BAA-894 genome.

**Figure 2:**
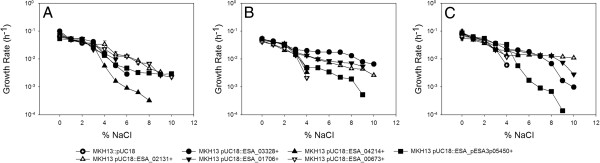
**Determination of the osmotolerance of *E. coli *** **MKH13 strains transformed with *proP *** **homologues from *C. sakazakii *** **BAA-894 in A) complex media, B) defined media supplemented with proline and C) defined media supplemented with betaine.**

Physiological analysis of *E.coli* MKH13 transformed with each *C. sakazakii proP* homologue reveals that six of the *proP* homologues initially identified *in silico* play a role in cellular osmoregulation in *E. coli* MKH13, a strain which is deficient in the compatible solute transporters putP, proP and proU rendering it sensitive to hyperosmotic conditions. Significant differences in growth rates were exhibited among strains grown in different environments where compatible solute availability varied. In particular, *E. coli* MKH13 expressing ESA_01706 failed to grow above 5% added NaCl when grown in LB (Figure 2), yet in minimal media with proline as the only available compatible solute this strain grew in the presence of up to 9% NaCl (growth rate of 0.005 h^-1^). If we consider that competitive inhibition among osmolytes has been demonstrated previously and the osmoprotective effects of osmolytes varies significantly then these results are to be expected [31,32]. Indeed, inhibition of glycine betaine uptake by other compatible solutes, or indeed glycine betaine analogues, is a phenomenon which has been observed previously [32]. Such structural similarities and differing osmoprotective ranges may offer a plausable explanation for the increased growth rates observed in this study in defined media (where there is only one compatible solute - proline or glycine betaine) in comparison to complex media rich in ‘competing’ osmoprotective compounds.

While many studies have previously focused on the importance of the ProP porter to *E. coli* osmotolerance, we are the first to demonstrate importance of ProP activity in the osmotolerance of the neonatal pathogen *C. sakzakii*. Indeed, each of the ProP porters analysed conferred osmotolerance when expressed in *E. coli* MKH13, albeit to varying degrees depending on compatible solute availability. Interestingly, glycine betaine appeared to be the most osmoprotective compound outperforming proline. Five of the strains expressing a *proP* homologue grew above 4% NaCl in betaine supplemented media in comparison to four strains demonstrating growth above 4% NaCl in proline supplemented media. Furthermore, growth rates of *E. coli* MKH13 expressing ESA_02131 and ESA_01706 were significantly higher in media supplemented with glycine betaine in comparison to proline (Table 3). Indeed it is likely that some of the transporters may be demonstrating specificity for one osmolyte over another but may transport both due to the structural similarities of proline and betaine. Another interesting finding was that ESA_00673 showed the highest osmotolerance of all the strains tested in LB, yet did not grow above 4% NaCl in defined media supplemented with either proline or glycine betaine suggesting that this porter might well be transporting an as yet unidentified osmolyte other than betaine or proline.

As outlined previously ESA_02131 was the most closely related homologue to the well characterized *proP* of *E. coli* and exhibited the characteristic C-terminal extension which is lacking in the other *C. sakazakii proP* homologues. The role of the carboxy-terminal extension in osmotolerance was defined by Culham et al. [33] when a derivative with alterations to specific amino acids on the C-terminal extension required a larger osmotic upshift for activation, when compared to the wild-type*.* The C-terminal domain has been implicated in osmosensing by other transporters such as BetP and OpuA in which osmolality controls the interaction of the C-terminal domain with each transporter, switching the transporter between active and inactive confirmations [34]. However, the C-terminal domain in ProP does not appear to be required for activation of the protein as orthologues without the coiled-coil domain of the C-terminal extension still function as osmosensory transporters [35]. That being said, research has shown that a higher osmolality is required to activate transporters lacking the coiled-coil [35]. The lack of this highly sensitive coiled-coil domain in the *C. sakazakii* ProP homologues may be a consequence of *C. sakazakii* existing in more extreme osmotic environments when compared to *E. coli* which depends on a single ProP which is highly tuned by the coiled coil. Indeed Tsataski et al. [35] have previously demonstrated that the osmotic activation of the transporter ProP of *E. coli* is tuned by its C-terminal coiled-coil and osmotically induced changes in the phospholipid composition.

## Conclusion

In the current study we have confirmed that six of the previously identified *proP* homologues play a role in the osmotolerance response of *C. sakazakii*. Gene expression analysis of each of these *proP* homologues demonstrated an increase in expression levels during an osmotic upshift. Furthermore, heterologous expression against an osmotically sensitive *E. coli* host revealed that each of the six homologues conferred an osmotolerance phenotype, albeit to varying degrees, in growth media with differences in osmolyte availability. The degeneracy of ProP porters in *C. sakazakii* goes some way to explaining the exteme osmotolerance phenotype of the bacterium, a factor which contributes significantly to its ability to survive in PIF. To date ProP in *E.coli* has been well characterized however this is the first study to demonstrate the importance of ProP in *C. sakazakii* osmotolerance. Knowledge of the transcriptional, translational and posttranslational regulation of these uptake systems will aid the future design and development of novel control stratagies for dealing with the pathogen, particularly in foods destined for neonatal consumption. Indeed, the use of small molecules to structurally mimic compatible solutes (smugglin technology) may be a viable alternative to antibiotics for the control of this pathogen in high risk foods such as powdered infant formula [36-38].

## Competing interests

The authors declare that they have no competing interests.

## Authors’ contributions

AF and CJ carried out the molecular cloning. RG participated in the real-time analysis. AF also carried out the physiological analysis and real-time analysis and drafted the manuscript together with RDS. All authors read and approved the final manuscript.
